# Effects of prenatal yoga on birth outcomes in nulliparous women: a systematic review and meta-analysis of randomized controlled trials

**DOI:** 10.1186/s12884-025-08279-4

**Published:** 2025-12-10

**Authors:** Feifei Chen, Hongjun Yu, Shuhua Peng

**Affiliations:** 1 Department of Nursing, Wuhan Donghu College, Wuhan, 435400 Hubei China; 2 Department of Nursing, Wuhan University of Science and Technology, Wuhan, 435400 Hubei China; 3 Department of Nursing, Xiaogan central hospital, Xiaogan, 435400 Hubei China

**Keywords:** Puerpera childbirth, Pregnancy, Yoga childbirth, Meta-analysis

## Abstract

**Background:**

Prenatal yoga uniquely integrates moderate aerobic activity with mindfulness‑based stress reduction, diaphragmatic breathing, and targeted pelvic‑floor conditioning. This study aimed to investigate whether yoga could influence the rates of cesarean section, episiotomy, perineal laceration, vaginal midwifery, preterm birth, and total labor time during delivery compared to standard nursing care.

**Method:**

Searches were conducted in MEDLINE, OVID, Scopus, PubMed, China Biology Medicine (CBM), Wiley, BMJ, JAMA, The Cochrane Library, Web of Science, VIP database, Wanfang database, CNKI, ClinicalTrials.gov, and the Cochrane Central Register of Controlled Trials, using a combination of keywords related to “prenatal yoga,” “pregnancy yoga,” “yoga birth RCT,” “pregnant woman yoga,” and pregnancy outcomes from database inception until December 2023. Bias assessment followed the Cochrane Handbook guidelines. The random-effects model calculated relative risk or mean differences with confidence intervals. Randomized controlled trials comparing yoga practice with general nursing exclusively during labor were included. Primary outcomes were rates of cesarean section, episiotomy, perineal laceration, vaginal midwifery, and preterm birth. Secondary outcomes included total labor time during delivery. Statistical analysis was performed using RevMan 5.4.

**Results:**

The meta-analysis included 14 randomized controlled trials with 3637 women. The control group received general nursing, while the experimental group underwent yoga classes and practice in addition to general nursing. The risk of bias was generally low. The rates of cesarean section (RR = 0.45, 95%CI: 0.38, 0.54, *P* < 0.01), vaginal delivery (RR = 0.66, 95%CI: 0.47, 0.93, *P* = 0.02), premature birth (RR = 0.29, 95%CI: 0.15, 0.56, *P* < 0.01), and perineal laceration (RR = 0.41, 95%CI: 0.25, 0.69, *P* < 0.0007) in the experimental group were lower than in the control group. Additionally, the total labor time for the experimental group was shorter than for the control group [MD=-2.10, 95%CI: -2.42, -1.79, *P* < 0.0001].

**Conclusions:**

Combining yoga practice with general nursing contributes to lower cesarean rates, shortened delivery times, reduced perineal lacerations, episiotomies, and preterm births.

**Supplementary Information:**

The online version contains supplementary material available at 10.1186/s12884-025-08279-4.

## Introduction

 Pregnancy is characterised by dramatic cardiovascular, musculoskeletal and neuro‑endocrine adaptations that can precipitate discomfort, anxiety and obstetric complications. Globally, operative deliveries continue to rise—cesarean section rates now exceed 30% in many countries—yet are not invariably associated with better neonatal or maternal outcomes. [1, 2] Throughout pregnancy, women often contend with various discomforts, including anxiety, fear, and depression. Mindfulness meditation and biofeedback have demonstrated effectiveness in enhancing mental well-being, addressing issues like depressive symptoms and anxiety [3]. 

Evidence‑based, low‑risk strategies that foster normal physiological birth are therefore a public‑health priority. Physical activity, encompassing practices like yoga, has proven beneficial in alleviating depressive symptoms and improving birth outcomes. Expressive writing has also shown success in postpartum applications and dealing with the challenges of pregnancy. Yoga, an ancient mind-body practice originating from India, is recognized as a holistic approach that addresses immune, neuromuscular, psychological, and pain conditions. [4, 5] The integrated nature of yoga involves adopting different postures, gradually controlling breathing to reduce the breath rate, and employing calming techniques such as meditation and chanting to enhance comfort during labor pain. Randomised trials show that moderate antenatal exercise lowers excessive gestational weight gain [2], improves glycaemic control [3] and reduces hypertensive disorders [4]. Prenatal yoga is a multimodal intervention that combines physical postures (āsana), diaphragmatic breathing (prāṇāyāma), mindfulness and pelvic‑floor engagement. Compared with generic aerobic exercise, yoga emphasises lumbopelvic alignment and relaxation responses that may translate into shorter first‑stage labour, lower instrumental birth and reduced analgesia use [5–7]. Neuroimaging studies also demonstrate attenuated hypothalamic‑pituitary‑adrenal activation after yoga practice, supporting its anxiolytic effect [8]. 

Two earlier syntheses reported favourable but heterogeneous effects of pregnancy yoga on labour duration and mode of delivery [9, 10]. However, both pooled ≤ 10 trials published before 2020 and lacked subgroup analyses by programme timing or duration. Since then, several high‑quality RCTs from diverse settings—including China, Iran and Australia—have been published, almost doubling the available sample size. Moreover, no meta‑analysis has systematically explored how initiation trimester or session frequency moderates effect estimates.

Based on this expanded evidence, our study aims to provide an updated, comprehensive synthesis of RCTs evaluating prenatal yoga’s impact on birth outcomes in nulliparous women. We further examine heterogeneity via meta‑regression and predefined sensitivity analyses. The findings will inform clinicians and policy‑makers seeking non‑pharmacological strategies to promote physiological childbirth.

## Methods

### Search strategy and study eligibility

The meta-analysis was reported following the Preferred Reporting Item for Systematic Reviews and Meta-Analyses (PRISMA) statement. A thorough and comprehensive investigation was conducted of English-language and Chinese‑language articles inception to 31 December 2023 referenced in the Pubmed, OVID, Scopus, Pubmed, China Biology Medicine (CBM), Wiley, BMJ, JAMA, The Cochrane Library, Web of science, VIP database, Wanfang database and CNKI, ClinicalTrials.gov, and the Cochrane Central Register of Controlled Trials. Yoga nursing intervention RCTs for pregnant women were included by screening headings and free words. Two reviewers (F.C and H.Y) obtained the following information from each of the included studies: the first author, year date of publication, study design, enrollment period, sample size, interventions, length of follow-up period. We used free text terms and MeSH terms in our search, which primarily including the following search terms: “prenatal yoga”, “pregnancy yoga”, “yoga birth RCT”, “pregnant woman yoga”, “expectant mother” AND yoga.

In terms of study design, only randomized controlled trials were included. Inclusion criteria: (1) Pregnant women with regular prenatal examination; (2) full-term singleton pregnancy with normal limbs; (3) no abnormal in extrapelvic measurements; (4) All the women were primiparas. (5) All the women were able to labor spontaneously; (6) All pregnant women signed informed consent. Exclusion criteria: (1) pregnant women with complications including diabetes mellitus, hypertension or other organ dysfunction; (2) poor compliance due to mental illness; (3) history of premature birth or abortion; (4) history of perineal surgery or vulvar inflammation. (5) those who withdrew the study. PICO eligibility criteria are summarised in Table S1.

### Data extraction and quality assessment

Two reviewers (F.C and H.Y) obtained the following information from each of the included studies: the first author, year date of publication, study design, enrollment period, sample size, delivery outcomes, interventions and selected articles according to the inclusion and exclusion criteria. The primary outcomes were cesarean section, degree of perineal tear, including first degree perineal tear and second degree perineal tear, and lateral perineal resection rate. The secondary outcomes included postpartum hemorrhage, total labor time, preterm birth, and perineal extension. If there are any disagreement, the third reviewer (S.P) would make the final decision or try to contact the author for supplement data.

The reviewers used Cochrane bias risk assessment tool to evaluate the quality of the included randomized controlled trials studies. The quality assessment and data extraction were undertaken using a standardized form. The quality of the studies was divided into three grades (ABC) according to the degree of eccentricity of the studies. The quality of the literature was evaluated by two researchers independently, and the third researcher negotiated and decided in case of disagreement.The international GRADE of recommendations assessment developmentand evaluation (GRADE) was used to evaluate the quality of the evidence. There were five factors that could reduce the quality of evidence: the limitations of the study, the inconsistency of the study results, the indirectness of the study results, the imprecision of the results, and publication bias.

### Statistical analysis

Statistical analyses Pooled RR and 95% CIs were calculated to examine the effect with differences in primary and secondary outcomes. The I_2_ statistic was used to examine the degree of heterogeneity among studies. A fixed effects model was used when there was significant heterogeneity (I_2_ < 50%); otherwise, a random effects model was employed. Funnel plots and publication bias was assessed by visual inspection of funnel‑plot symmetry and Egger’s regression test (two‑tailed, α = 0.05) conducted in Stata 17 (StataCorp, College Station, TX) [11]. Sensitivity analyses were conducted to examine the robustness of pooled estimates. We applied four approaches: (i) leave‑one‑out influence analysis, sequentially omitting each study to assess its impact on the pooled effect; (ii) comparison of studies at low versus moderate risk of bias (RoB); (iii) subgrouping by yoga‑programme initiation (early ≤ 20 weeks vs. late > 20 weeks gestation); and (iv) subgrouping by programme duration (short < 8 weeks vs. long ≥ 8 weeks). For each approach, pooled RRs/MDs and 95% CIs were recalculated using the same fixed‑ or random‑effects model criteria described above. Influence analysis was implemented in RevMan 5.4, while subgroup and meta‑regression analyses were performed in Stata 17 using the ‘metan’ and ‘metareg’ commands. Methods follow recommendations outlined by Borenstein et al. [12] RevMan 5.4 software was used to prepare and maintain reviews from the Cochrane database, and the statistical analysis. Statistical significance was defined as *p* < 0.05.

## Results

### Study characteristics

**Fig. 1:**
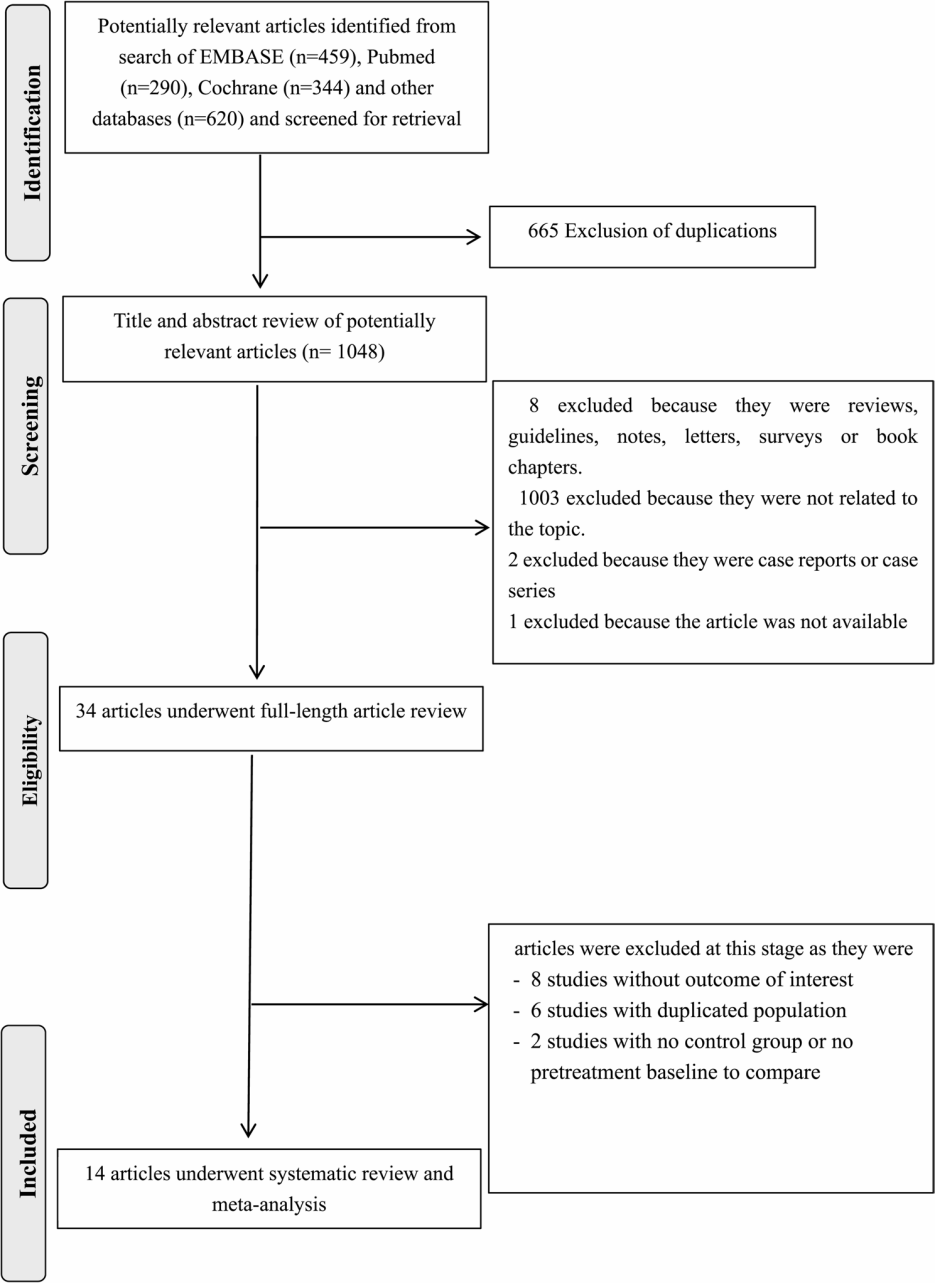
The flowgram of inclusion of studies

The retrieval process yielded a total of 793 relevant articles initially. After excluding 664 articles (314 due to repetition, 176 through title and abstract review, and 167 unrelated to pre-pregnancy yoga), 34 articles remained. Upon thorough examination of the full texts, 19 articles were further excluded for not meeting the inclusion criteria (10 non-controlled trials and 6 articles published before 2015). Consequently, 14 articles were ultimately included in the analysis. All the studies incorporated were randomized controlled trials, comprising 11 Chinese articles and 4 English articles, involving a total of 3637 subjects, as depicted in Fig. 1.

**Fig. 2:**
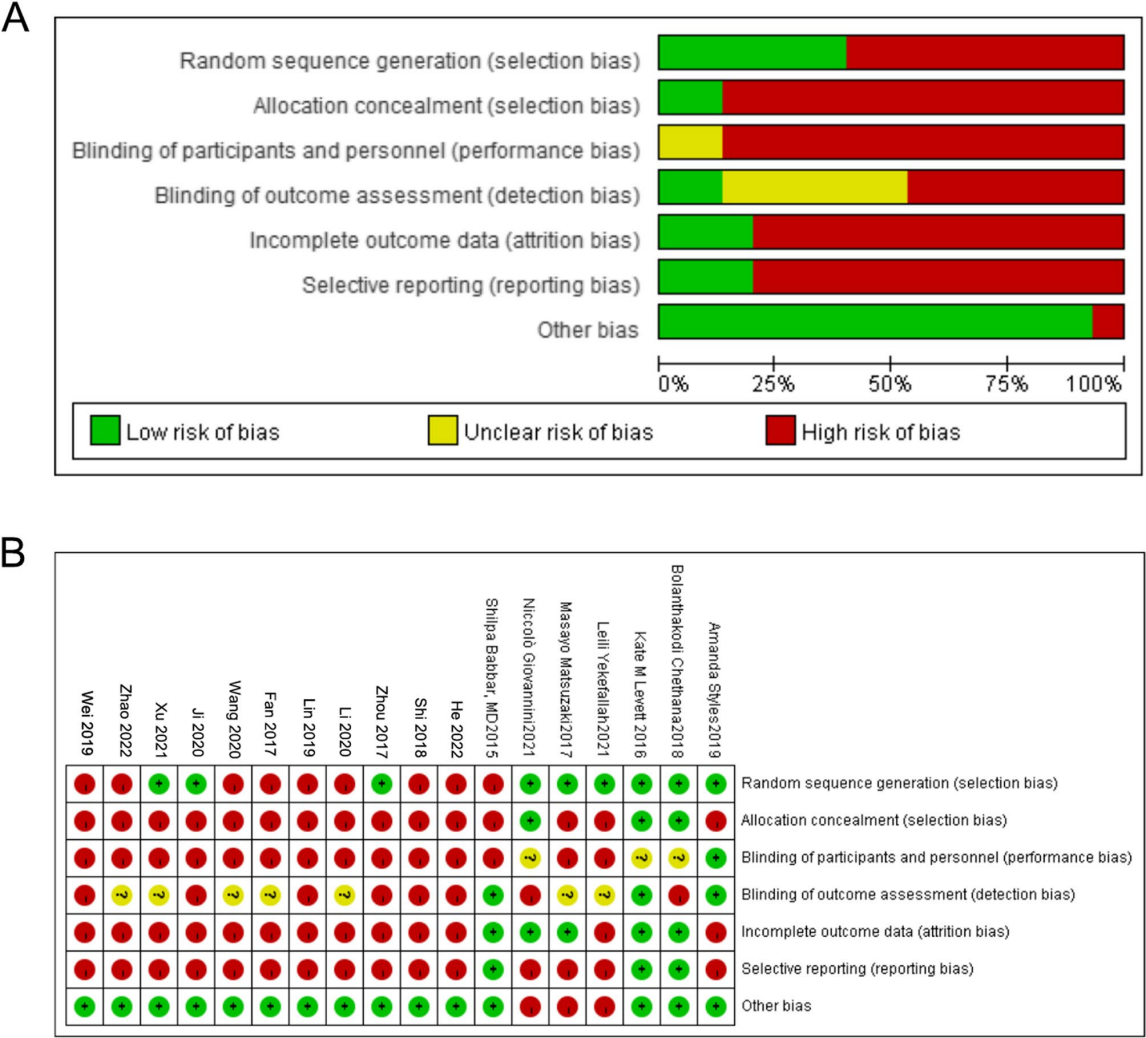
**A** Risk analysis of bias in clinical studies on the impact of prenatal yoga on delivery outcomes; (**B**) Summary of bias risks in clinical studies on the impact of prenatal yoga on delivery outcomes

The 14 included articles are detailed in Table 1, with 13 mentioning randomness [6–13, 15–20]. Among these, 2 studies [15, 18] followed the random number table method, 1 study [6] utilized block randomized parallel control, 1 study [6] employed odd and even numbers on the maternal perinatal health care card, 1 study [8] randomized via a double color ball, 1 study [9] used computer randomization, and 7 study [5, 7, 10, 14, 16, 19]– [20] only made a general mention of randomness without specifying the method. Notably, the specific grouping method was unclear in these cases. Two studies didn’t use the term “random” (Table 1), and the bias risk assessment results are depicted in Fig. 2A and B. Due to the requirement for pregnant women to engage in prenatal yoga, achieving blinding was challenging, leading to a higher risk of bias [13, 14].

**Table 1:** Basic characteristics of the included literature

Authors	Year	Type of research	Sample size	Intervention methods		Outcome measures	Literature quality
			Experimental group/control group	Experimental group	Control group		
Leili Yekefallah et al. [8]	2021	RCT	35/35	75 min yoga class twice a week at 26–27 weeks gestation	Routine care	126	A
Bolanthakodi et al. [31]	2018	RCT	67/58	After 30 weeks of gestation, perform prenatal yoga training	Routine care	56	A
Kate M Levett et al. [7]	2016	RCT	88/83	Prenatal yoga class education with breathing at 24–36 weeks of gestation	Routine care	234	B
Shilpa Babbar, et al. [9]	2015	RCT	23/23	Professional yoga instructors perform one-on-one prenatal yoga exercises between 28 and 37 weeks of gestation	Routine care	2	B
He Xiaoqing et al. [10]	2022	RCT	30/30	Before 12 weeks of gestation, do breathing exercises; And yoga after 13 weeks	Routine care	234	B
Shuang Shi [5]	2018	RCT	298/298	Pregnant women began to participate in pregnancy yoga classes at 16 weeks of gestation, each class was 60 min, three times a week, and ended at 36 weeks of gestation	Routine care	1236	B
Zhou Ru et al. [6]	2017	RCT	186/189	From the 16th week of pregnancy, under the professional guidance of a yoga teacher, yoga exercise was performed twice a week, 60 min each time	Routine care	139	B
Lin Wei [13]	2019	RCT	60/60	Training starts at 16 weeks of gestation for pregnant women, including abdominal deep breathing exercises, mountain exercises, etc.	Routine care	124	B
Fan Rong [17]	2017	RCT	80/80	Add yoga training after 16 weeks of pregnancy	Routine care	24568	B
Wang Liye et al. [14]	2020	RCT	55/55	Increase pregnancy yoga training, primiparas can be trained after admission, 2 times/week, 60 min/time, training until delivery	Routine care	5	B
Chi Peng Hui [18]	2020	RCT	50/50	Pregnant women come to the hospital regularly to receive prenatal education, attend pregnant women’s school, explain the daily diet, exercise program, and assess the condition of the perineum	Routine care	1235	B
Xu Xin et al. [15]	2021	RCT	30/30	30 Instruct pregnant women to start yoga ball training at 7 months of gestation	Usual care	245	B
Zhao Qi et al. [19]	2022	RCT	75/75	From the 15th week of pregnancy, the nursing staff guided the pregnant women to do yoga training, select their favorite yoga training postures, and train for 30 min every day	Routine care	1235	B
Wei Xin [32]	2019	RCT	30/30	Exercise with different yoga methods in early, middle and late pregnancy	Usual care	25	B

1. Episiotomy rate 2. Cesarean section rate 3. Perineal laceration rate 4. Assisted vaginal delivery rate 5. Total duration of labor 6. Preterm birth rate 7. Vital signs 8. Postpartum hemorrhage 9. Perineal elasticity and perineal length at delivery

### Efficacy of prenatal yoga on delivery mode

**Fig. 3:**
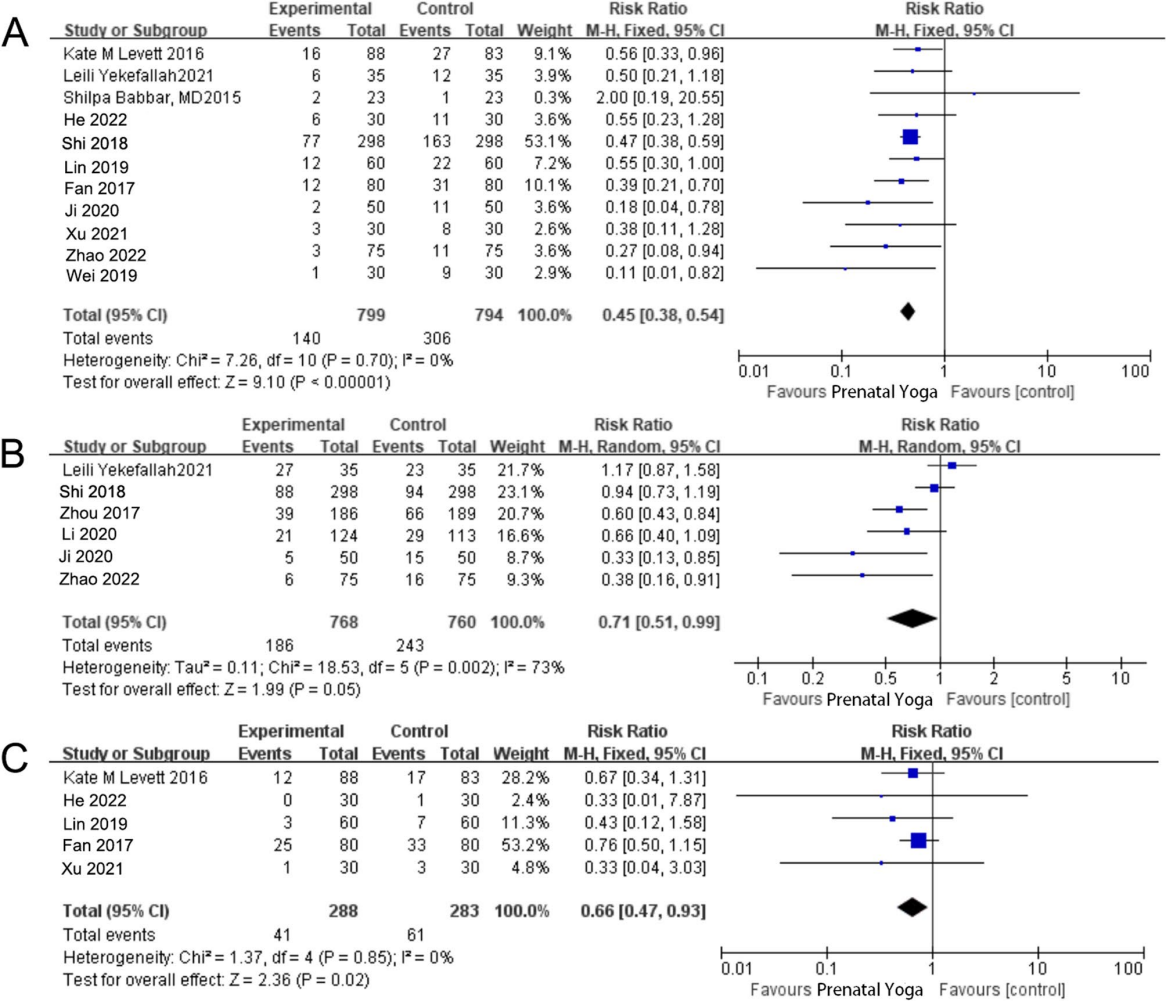
**A** The forest map for comparison of cesarean section; (**B**) The forest plot for comparison of total vaginal lateral resection rates; (**C**) The forest plot for comparing vaginal delivery rates

**Fig. 4:**
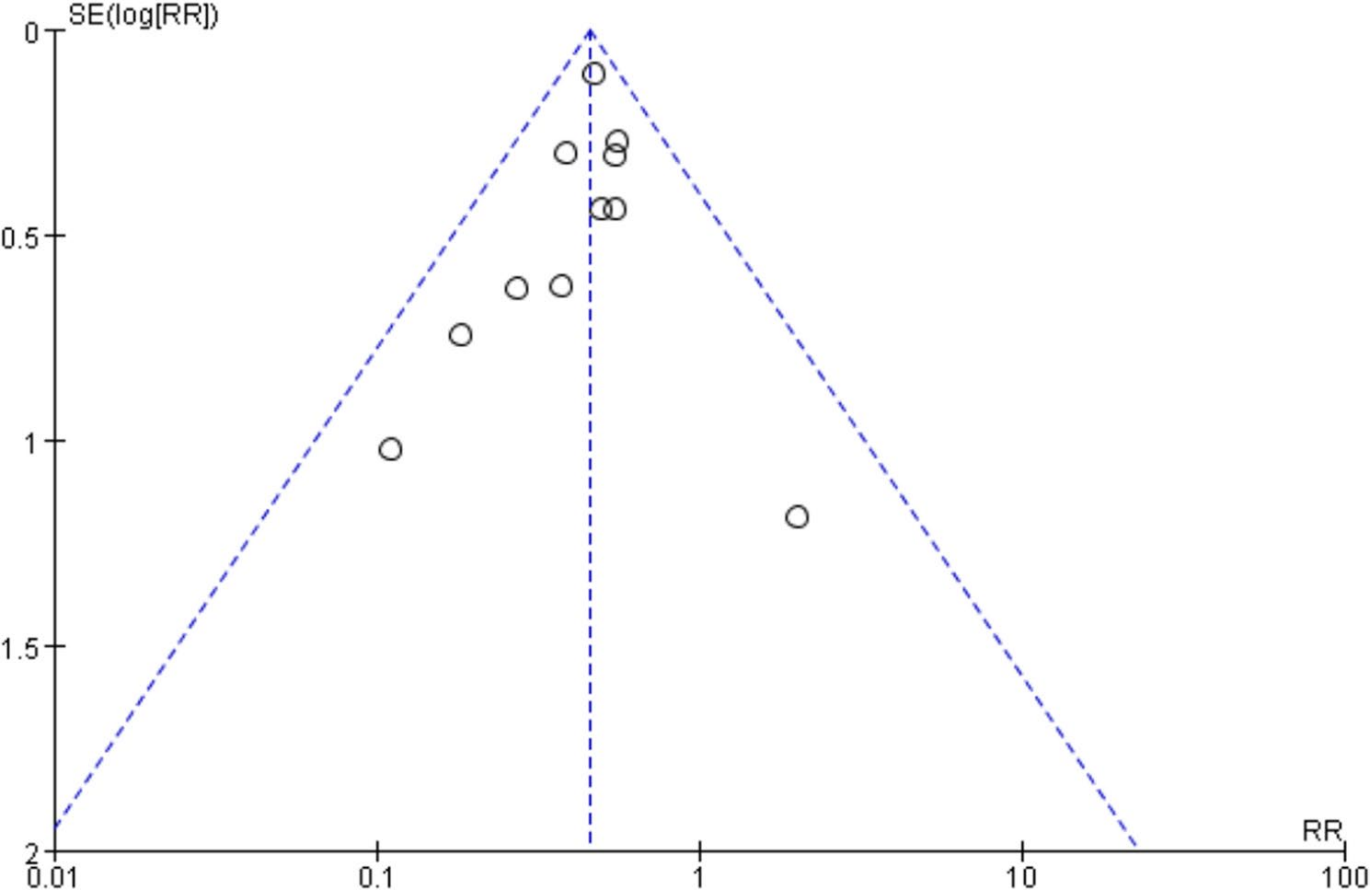
Funnel plot assessing publication bias for the cesarean section outcome

11 studies [5, 7–10, 13]– [15, 17–20] compared cesarean section rates between the two groups. The heterogeneity test indicated good consistency among the studies (I^2^ = 0%, *P* = 0.70), leading to the adoption of a fixed-effect model. The analysis results revealed a lower cesarean section rate in the observation group compared to the control group [RR = 0.45, 95% CI (0.38, 0.54), *P* < 0.01], demonstrating minimal bias, as depicted in Figs. 3A and 4.

### Efficacy of prenatal yoga on episiotomy rates

The impact of prenatal yoga on episiotomy rates was assessed in six studies [6, 8, 10, 16, 18, 19]. The heterogeneity test revealed substantial variation among the studies (I^2^ = 73%, *P* < 0.01). A sensitivity analysis of the meta-analysis results for this outcome index found no identifiable source of heterogeneity, prompting the use of a random-effects model [RR = 0.71, 95% CI (0.51, 0.99), *P* = 0.05]. However, the difference did not achieve statistical significance. The forest plot results are illustrated in Fig. 3B, with Z = 1.99 and *P* = 0.05. Based on the overall episiotomy count, there is insufficient evidence to support the notion that prenatal yoga effectively reduces the incidence of episiotomy.

### Efficacy of prenatal yoga on mode of delivery

The impact of prenatal yoga on the mode of delivery was investigated in five studies [7, 10, 13, 15, 17], comparing the vaginal delivery rates between the two groups. The heterogeneity test indicated strong consistency among the studies (I^2^ = 0%, *P* = 0.85), leading to the utilization of a fixed-effect model. The analysis results revealed a lower rate of vaginal midwifery in the observation group compared to the control group [RR = 0.66, 95% CI (0.47, 0.93), *P* = 0.02], as depicted in Fig. 3C.

### Efficacy of prenatal yoga on perineal laceration rate

**Fig. 5:**
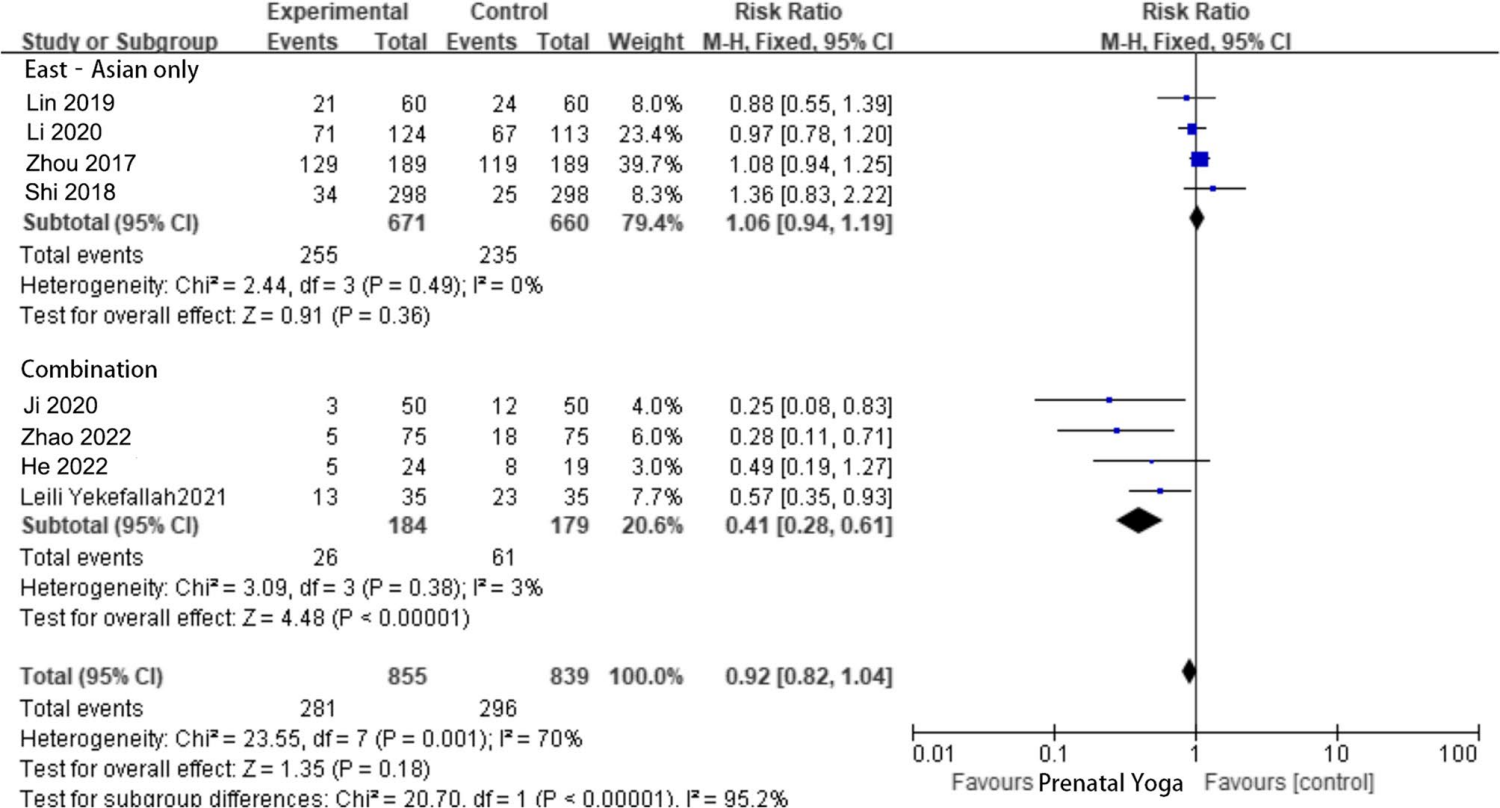
The subgroup analysis for comparing perineal laceration rates

The impact of prenatal yoga on the perineal laceration rate was assessed in nine studies. [7, 8, 10–13, 16, 19] The test revealed heterogeneity, with I^2^ = 69%, *P* < 0.01. Sensitivity analysis identified Kate et al.‘s study [7] as a significant source of heterogeneity. After its exclusion, the studies were subgrouped based on heterogeneity, and further analysis was conducted. The East‑Asian only subgroup ^[5~6, 13~14]^ showed no heterogeneity (I^2^ = 0%, *P* = 0.49), and the results [RR = 1.06, 95% CI (0.94, 1.19), *P* = 0.36] were not statistically significant. The Combination subgroup (Asian, Iran, India, Australia) [8, 10, 18, 19] exhibited low heterogeneity (I^2^ = 3%, *P* = 0.38), and the result [RR = 0.41, 95% CI (0.28, 0.61), *P* < 0.0001] was statistically significant, as depicted in Fig. 5.

### Efficacy of prenatal yoga on the rate of first-degree perineal laceration

**Fig. 6:**
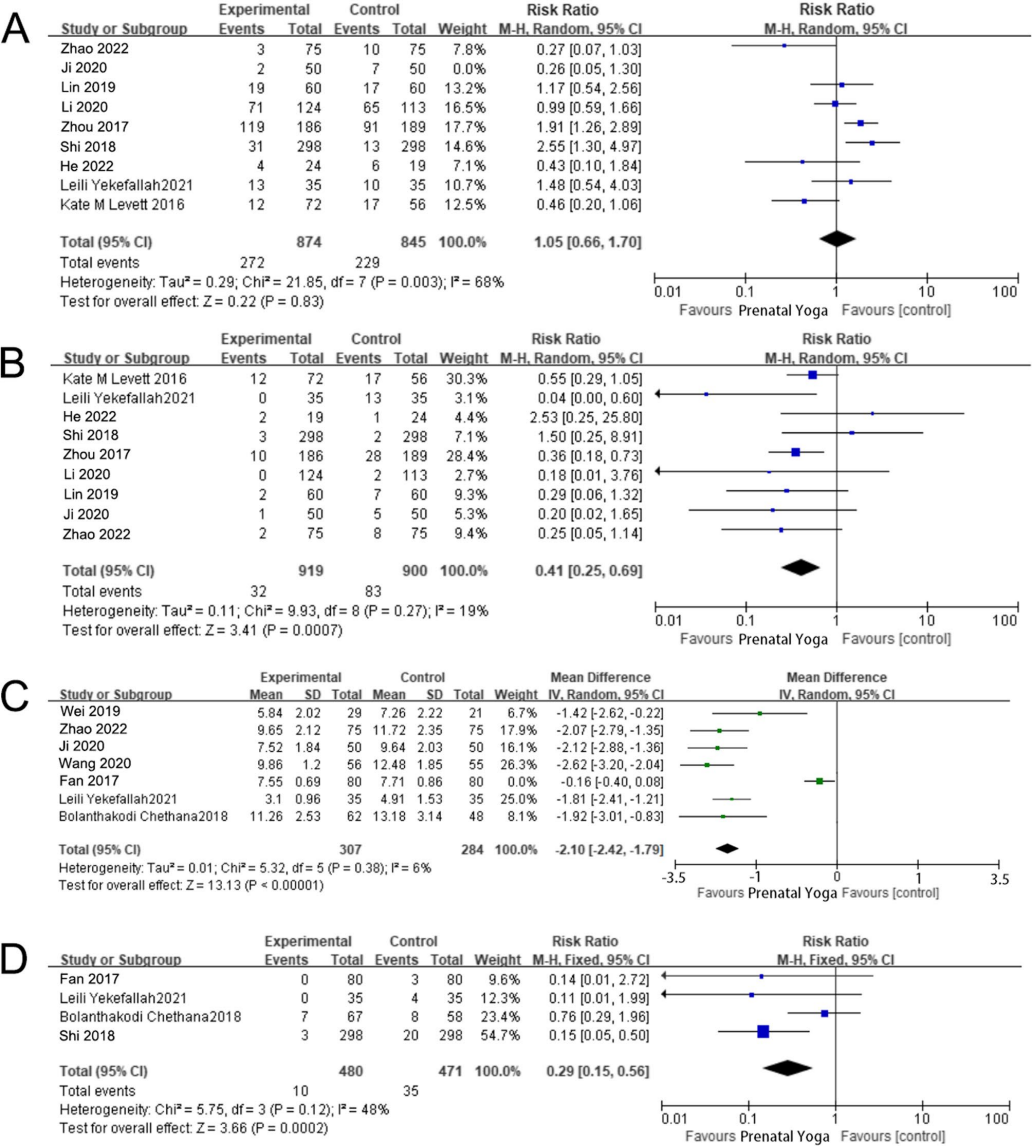
**A** The forest map for comparing the rates of perineal lacerations; (**B**) The forest map for comparing the rates of 2nd degree perineal lacerations; (**C**) The forest diagram for comparing the total delivery time; (**D**) The forest plot for comparing premature birth rates in newborns

The impact of prenatal yoga on the rate of first-degree perineal laceration was examined in nine studies. [7, 8, 10–13, 16, 18, 19] A considerable heterogeneity among the studies was revealed by the heterogeneity test (I_2_ = 68%, *P* = 0.003). Despite conducting a thorough sensitivity analysis, no specific source of heterogeneity was identified. Consequently, the random-effects model was employed [RR = 1.05, 95% CI (0.66, 1.70), *P* = 0.83], and the observed difference was not statistically significant. The forest plot illustrating these results is depicted in Fig. 6A.

The impact of prenatal yoga on the rate of first-degree perineal laceration was investigated through a comparison of nine studies on second-degree perineal laceration between two groups. The heterogeneity test indicated a good consistency among the studies (I^2^ = 19%, *P* = 0.27), leading to the utilization of a fixed-effect model. The analysis results demonstrated that the rate of second-degree perineal laceration in the observation group was significantly lower than that in the control group [RR = 0.41, 95% CI (0.25, 0.69), *P* < 0.0007], as illustrated. in Fig. 6B.

### Efficacy of prenatal yoga on total labor time

The impact of prenatal yoga on total labor time was assessed through the analysis of six studies [6, 14, 17–19] that measured the labor duration for parturient women. The heterogeneity test revealed significant variability among the studies (I^2^ = 95%, *P* < 0.01). Sensitivity analysis identified Fan Rong’s study [17] as a major contributor to the heterogeneity. After excluding this study and conducting another heterogeneity test, the remaining five studies exhibited no significant heterogeneity (I^2^ = 6%<50%, *P* < 0.01, *P* = 0.38 > 0.1). The fixed-effect model was employed for the meta-analysis, yielding results indicating a reduction in total labor time [MD=−2.10, 95% CI (−2.42, −1.79), *P* < 0.0001], as illustrated in Fig. 6C.

### Efficacy of prenatal yoga on maternal delivery

The impact of prenatal yoga on maternal delivery was assessed through four studies comparing the preterm birth rate of newborns between the two groups. The heterogeneity test yielded I^2^ = 48%, *P* = 0.12, leading to the application of the fixed-effect model. Results indicated a lower episiotomy rate in the observation group compared to the control group [RR = 0.29, 95% CI (0.15, 0.56), *P* = 0.0002 < 0.01], as depicted in Fig. 6D.

### Evaluation of study evidence and meta‑regression

The GRADE Profiler 3.6 software was employed to assess the evidence for each outcome indicator, as outlined in Table 2. The evidence grading for outcome indicators in the prenatal yoga observation group versus the control group during pregnancy resulted in an intermediate grade for the cesarean section rate. The rates of perineal laceration and preterm birth were both classified as low. Episiotomy, vaginal delivery, and the total duration of labor received an extremely low evidence grade. The Egger’s regression asymmetry test P value was 0.156 and no publication bias with *P* > 0.05 for all analyses.

**Table 2:** GRADE evidence evaluation of included studies

Variables	Number of articles	Assessment of evidence quality					Number of cases		Number of cases		Evidence quality	Importance
		Limitations	Inconsistency	Indirectness	Imprecision	Bias of publication	Observation group	Conventional group	RR/MD	95%CI		
Cesarean rate	11	2^a^	0	0	0	0	799	794	0.45	[0.38,0.54]	Median	Yes
Episiotomy rate	6	2^a^	1^d^	0	0	1^f^	768	760	0.71	[0.51,0.99]	Very low	Yes
Perineal laceration rate	9	2^a^	1^d^	0	0	0	924	895	0.85	[0.70,1.04]	Low	Yes
Vaginal midwifery rates	5	2^a^	0	0	1^e^	1^f^	288	283	0.66	[0.47,0.93]	Very low	Yes
Total labor time	5	2^a^	2^c^	0	1^e^	1^f^	352	329	−1.71	[−2.81, −0.61]	Very low	Yes
Preterm birth rate	4	2^a^	0	0	0	1^f^	480	471	0.29	[0.15,0.56]	Low	Yes

RCT randomized controlled trial: 0 indicated no downgrade, −1 indicated one downgrade, −2 indicated two down grade. a means the included study had two or more high risks of bias in terms of randomization, blinding, allocation concealment, completeness of outcome data or selective reporting; b means the included study had one high risk of bias in terms of randomization, blinding, allocation concealment, completeness of outcome data or selective reporting; c means the included study 75% ≤ I_2_ ≤ 100%; d is 50% ≤I_2_ < 75% of included studies; e is small sample size (< 400 for continuous variables, < 300 for dichotomous variables) or the 95% confidence interval (CI) crossing the null line or the effect size was [0.75–1.25]; f is asymmetric funnel plot or the number of included studies was less than 9 or all of them had positive results

Meta‑regression using programme duration (6–12 weeks) as a continuous moderator revealed a significant inverse association for overall perineal‑laceration rate (slope β = −0.07 ± 0.03 logRR per week, *P* = 0.04), explaining 21% of between‑study variance (Figure S1). For episiotomy and first‑degree laceration, slopes were small and non‑significant (β = −0.02 ± 0.03, *P* = 0.46 and β = −0.01 ± 0.04, *P* = 0.78, respectively; Figures S2–S3). These findings suggest that longer prenatal‑yoga programme may confer greater protection against overall perineal trauma but have little impact on episiotomy or superficial lacerations.

## Discussion

This meta-analysis aims to evaluate the effects of yoga during pregnancy on the delivery outcomes of pregnant women. Among the 14 RCTs included, the quality evaluation results of 2 articles were Grade A, and the quality evaluation results of 13 articles were Grade B. Quality issues identified in the literature include the incomplete explanation of the random sequence generation. Of the 14 included studies, 13 [6–12, 14–20] literatures mentioned the word random, but only 6 [6, 8, 9, 15, 18] literatures explained the specific grouping method, 7 [5, 7, 10, 14, 16, 19, 20] literatures only mentioned the word random, but did not describe the specific grouping method, and 2 [13, 14] literatures did not mention the word random. Allocation and concealment were only mentioned in 2 articles [6, 7], and the remaining articles did not mention it. It was difficult to implement the blinding of mothers and staff, 2 studies [6, 7] were single blind, and 13 studies [8–20] did not involve blinding. Overall, outcome data integrity and bias control were high, and data heterogeneity was acceptable, enhancing the credibility of the study results.

Yoga during pregnancy demonstrated a positive effect in reducing the rates of cesarean section and vaginal midwifed delivery. [7–13, 15–21] The application of yoga during pregnancy not only reduces cognitive pressure and anxiety but also moderately decreases the cesarean section rate, enhancing pregnant women’s satisfaction, aligning with the study results. [22, 23] [24] The purpose of yoga training is to improve limb coordination and personal balance, increasing pelvic floor muscle tension and strengthening abdominal and back muscles. This minimizes damage to the pelvic floor during abdominal cavity enlargement, improves pelvic state, enhances ligament toughness, and facilitates fetal delivery during childbirth. The increased rate of natural delivery also contributes to reduced vaginal damage. Free-body yoga training proves effective in reducing cesarean section and vaginal midwifery rates, minimizing maternal injuries.

Yoga exhibits beneficial effects on pregnancy-related changes and pain, significantly reducing the preterm birth rate in newborns [6, 8, 17]. Evidence suggests that regular exercise and pregnancy yoga during pregnancy, particularly at moderate to heavy intensity, can lower the risk of preterm birth. [21, 25, 26] The analysis attributes these benefits to yoga helping pregnant women prepare for childbirth, alleviating back pain, effectively supporting extra weight, improving blood circulation, reducing blood pressure and heart rate, and enhancing metabolic output for fetal development and nutrient absorption. By relieving muscle tension, increasing flexibility, muscle strength, endurance, and energy, yoga contributes to a more comfortable pregnancy, enhancing adaptability and decreasing premature contractions [27]. Ultimately, yoga exercise reduces premature birth and intrauterine growth restriction, fostering healthier fetal development in the uterus.

Yoga exercise in this study significantly reduces the risk of perineal trauma. [7, 10–12, 16, 18, 19] Prenatal yoga practice benefits women experiencing anxiety, depression, stress, low back pain, and sleep disorders, resulting in improved pregnancy outcomes and decreased perineal lacerations. [28, 29] The analysis attributes these benefits to yoga, especially pelvic floor muscle exercises, increasing maternal flexibility and perineal extension. This helps mothers fully utilize perineal extension during childbirth, reducing the occurrence of second-degree perineal lacerations. Additionally, yoga promotes maternal cooperation with midwives, minimizing perineal lacerations.

Corrigan et al. (2022) conducted a systematic review of 14 RCTs that partly overlap with our evidence base. They found a modest but significant reduction in overall labour duration yet reported no clear effect on cesarean section or episiotomy rates. By contrast, our updated meta‑analysis—which incorporates three recent East‑Asian trials published after Corrigan’s search cut‑off (Zhao 2022; Ji 2020; Leili 2021)—confirms the benefit on labor duration (MD = 2.10 h) and demonstrates significant reductions in cesarean section (RR = 0.45) and perineal trauma (RR = 0.41). Both reviews highlight moderate study quality and heterogeneity; however, our meta‑regression indicates programme duration (> 8 weeks) explains part of the between‑study variance, a moderator not explored by Corrigan et al. Taken together, the two syntheses suggest that well‑structured, sufficiently long prenatal‑yoga programme can enhance physiological birth, while emphasizing the need for larger, blinded multicenter RCTs to confirm effects across diverse settings.

The study results indicate that pregnancy yoga can reduce total labor time on a certain extent. This reduction may be attributed to pregnant women effectively managing pain and increasing pain tolerance through yoga breathing techniques and uterine contractions. Yoga exercise strengthens the levator ani muscle, improving contraction strength compared to women who do not practice yoga. [6, 14–21, 24, 30] This, in turn, enhances perineum malleability and length, indirectly promoting fetal delivery and shortening the labor process. [26, 27] Overall, pregnancy yoga proves to be a non-invasive, easy-to-learn psychosomatic medicine and complementary health practice, effectively reducing labor pain and potentially improving labor outcomes.

This study systematically evaluates the effects of yoga during pregnancy on maternal delivery outcomes, affirming its positive impact. Prenatal yoga offers numerous benefits throughout pregnancy, including shortened labor time, increased comfort, support for natural vaginal delivery, and decreased rates of cesarean section and preterm birth. Nurses and midwives can enhance their professional quality and health education skills, advocating for the promotion and application of yoga during pregnancy. However, considering the influence and limitations of the included literature, these conclusions require verification through more high-quality, multi-center, and large-sample RCTs to provide robust evidence-based support.

## Conclusion

Our study systematically evaluates the effects of yoga during pregnancy on maternal delivery outcomes, affirming its positive impact. Prenatal yoga offers numerous benefits throughout pregnancy, including shortened labor time, increased comfort, support for natural vaginal delivery, and decreased rates of cesarean section and preterm birth.

## Supplementary Information

Supplementary Material 1.

Supplementary Material 2.

Supplementary Material 3.

Supplementary Material 4.

Supplementary Material 5.

Supplementary Material 6.

## Data Availability

All data generated or analyzed during this study are included in the published article.
