# Supplementary material for: Effects of prenatal yoga on birth outcomes in nulliparous women: a systematic review and meta-analysis of randomized controlled trials
Source: BMC Pregnancy Childbirth. 2025 Dec 10;25:1302. doi: 10.1186/s12884-025-08279-4 (PMC12699887; doi:10.1186/s12884-025-08279-4)
Supplement: Supplementary file 2 — Supplementary Material 2. [file 12884_2025_8279_MOESM2_ESM.docx]

eTable 1. Search Strategy details

Pubmed

| # | Searches | Results |
| --- | --- | --- |
| 1 | Search pregnancy [mh] | 1,019,105 |
| 2 | Search pregnancy [Title/Abstract] | 487,777 |
| 3 | Search (((((((prenatal [Title/Abstract]) OR birth [Title/Abstract]) OR pregnant woman [Title/Abstract]) OR expectant mother [Title/Abstract]) OR woman pregnant [Title/Abstract]) OR Obstetric Labor [Title/Abstract]) OR Pregnancies [Title/Abstract] OR Gestation [Title/Abstract]) | 606,883 |
| 4 | Search (1 or 2 or 3) | 1,368,504 |
| 5 | Search Yoga [mh] | 3,895 |
| 6 | Search (((Yoga [Title/Abstract]) OR orthodox system of Hindu philosophy [Title/Abstract]) OR raja-yoga) | 7,011 |
| 7 | Search (5 or 6) | 7,503 |
| 8 | Search (4 and 7) | 290 |

Embase

| # | Searches | Results |
| --- | --- | --- |
| 1 | ' pregnancy '/exp | 894,341 |
| 2 | ' prenatal ':ab,ti OR ' birth ':ab,ti OR ' pregnant woman ':ab,ti OR ' expectant mother ':ab,ti OR ' woman pregnant ':ab,ti OR ' Obstetric Labor ':ab,ti OR ' Pregnancies ':ab,ti OR ' Gestation ':ab,ti | 798,726 |
| 3 | 1 or 2 | 1,383,838 |
| 4 | ' Yoga '/exp | 11,818 |
| 5 | ' Yoga ':ab,ti OR ' orthodox system of Hindu philosophy ':ab,ti OR ' raja-yoga ':ab,ti | 9,911 |
| 6 | 4 or 5 | 13,831 |
| 7 | 3 AND 6 | 459 |

Cochrane Library

| # | Searches | Results |
| --- | --- | --- |
| 1 | [mh " pregnancy "] | 76,707 |
| 2 | (prenatal OR birth OR pregnant woman OR expectant mother OR woman pregnant OR obstetric Labor OR pregnancies OR gestation):ti,ab,kw | 67,643 |
| 3 | #1 or #2 | 104,951 |
| 4 | [mh " yoga "] | 5,738 |
| 5 | (yoga or orthodox system of Hindu philosophy or raja-yoga):ti,ab,kw | 5,394 |
| 6 | #4 or #5 | 5,738 |
| 7 | animal*:ti,ab,kw | 38,970 |
| 8 | human*:ti,ab,kw | 1,271,430 |
| 9 | #7 not #8 | 5,621 |
| 10 | #3 and #6 | 345 |
| 11 | #10 not #9 | 344 |

ClinicalTrials.gov

| # | Searche Conditions | Results |
| --- | --- | --- |
| 1 | (yoga OR orthodox system of Hindu philosophy OR raja-yoga) AND (prenatal OR birth OR pregnant woman OR expectant mother OR woman pregnant OR Obstetric Labor OR Pregnancies OR Gestation) | 0 |
