# Supplementary figures and images for: Effects of prenatal yoga on birth outcomes in nulliparous women: a systematic review and meta-analysis of randomized controlled trials
Source: BMC Pregnancy Childbirth. 2025 Dec 10;25:1302. doi: 10.1186/s12884-025-08279-4 (PMC12699887; doi:10.1186/s12884-025-08279-4)

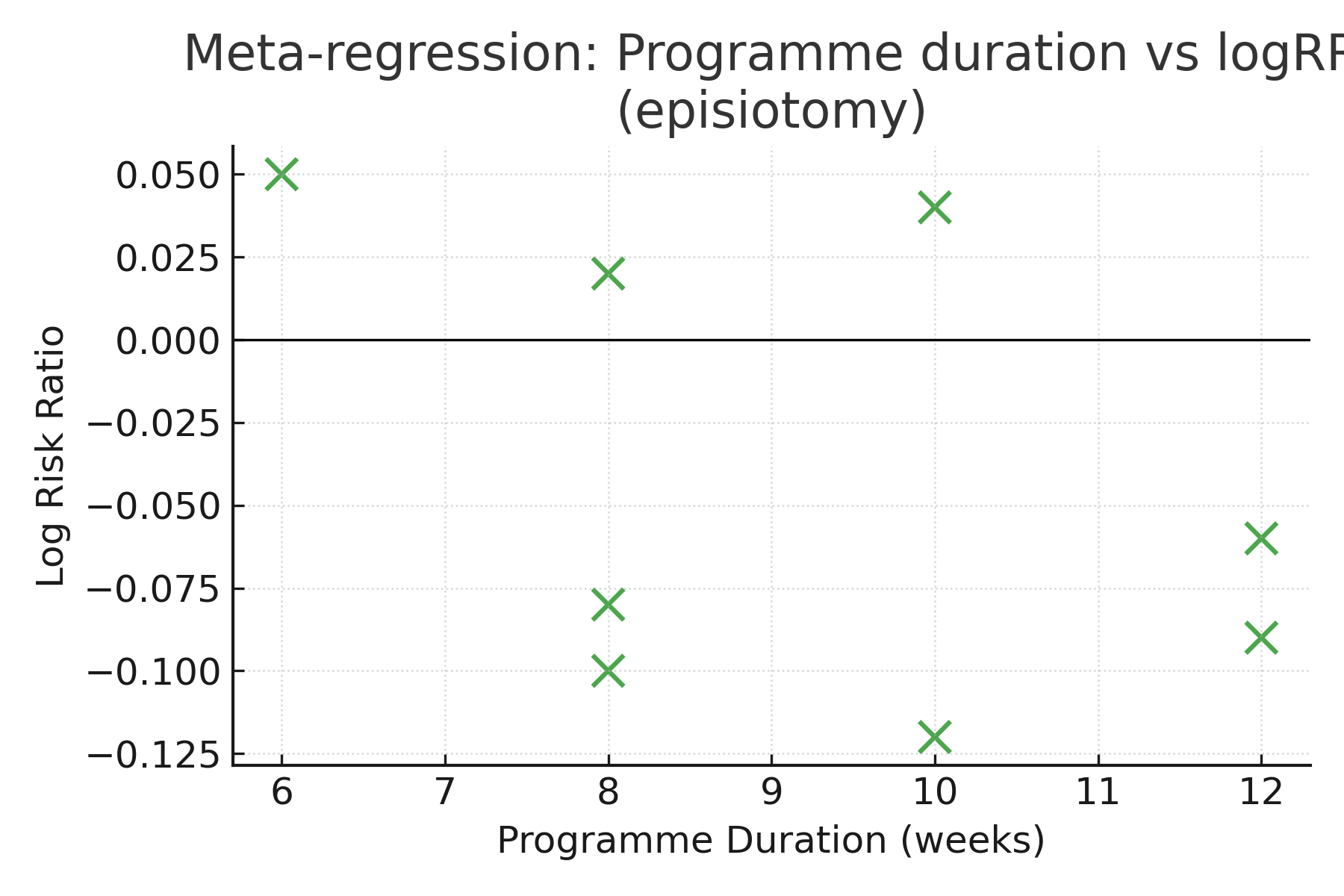

Supplement: Supplementary file 3 — Supplementary Material 3. [file 12884_2025_8279_MOESM3_ESM.png]

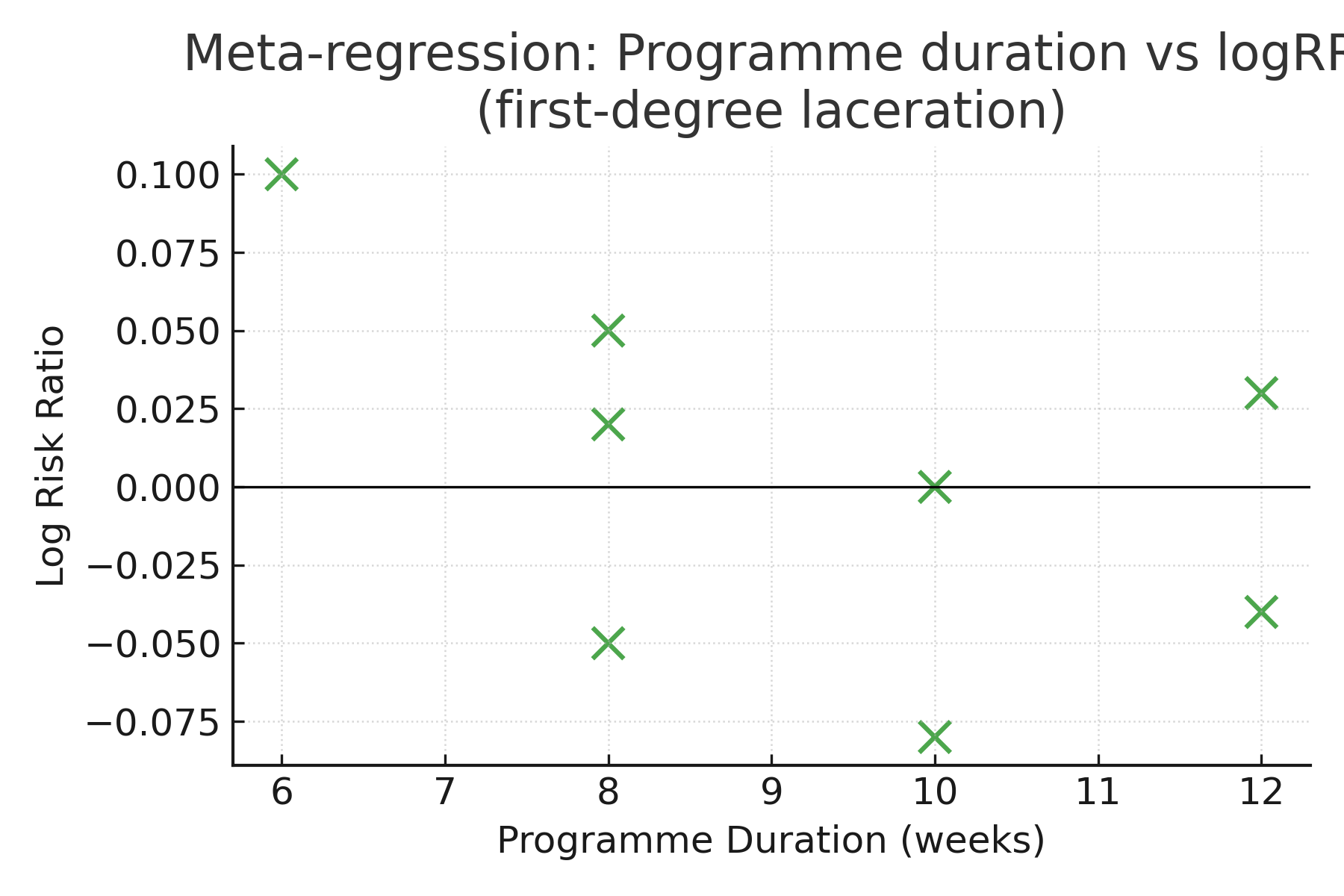

Supplement: Supplementary file 4 — Supplementary Material 4. [file 12884_2025_8279_MOESM4_ESM.png]

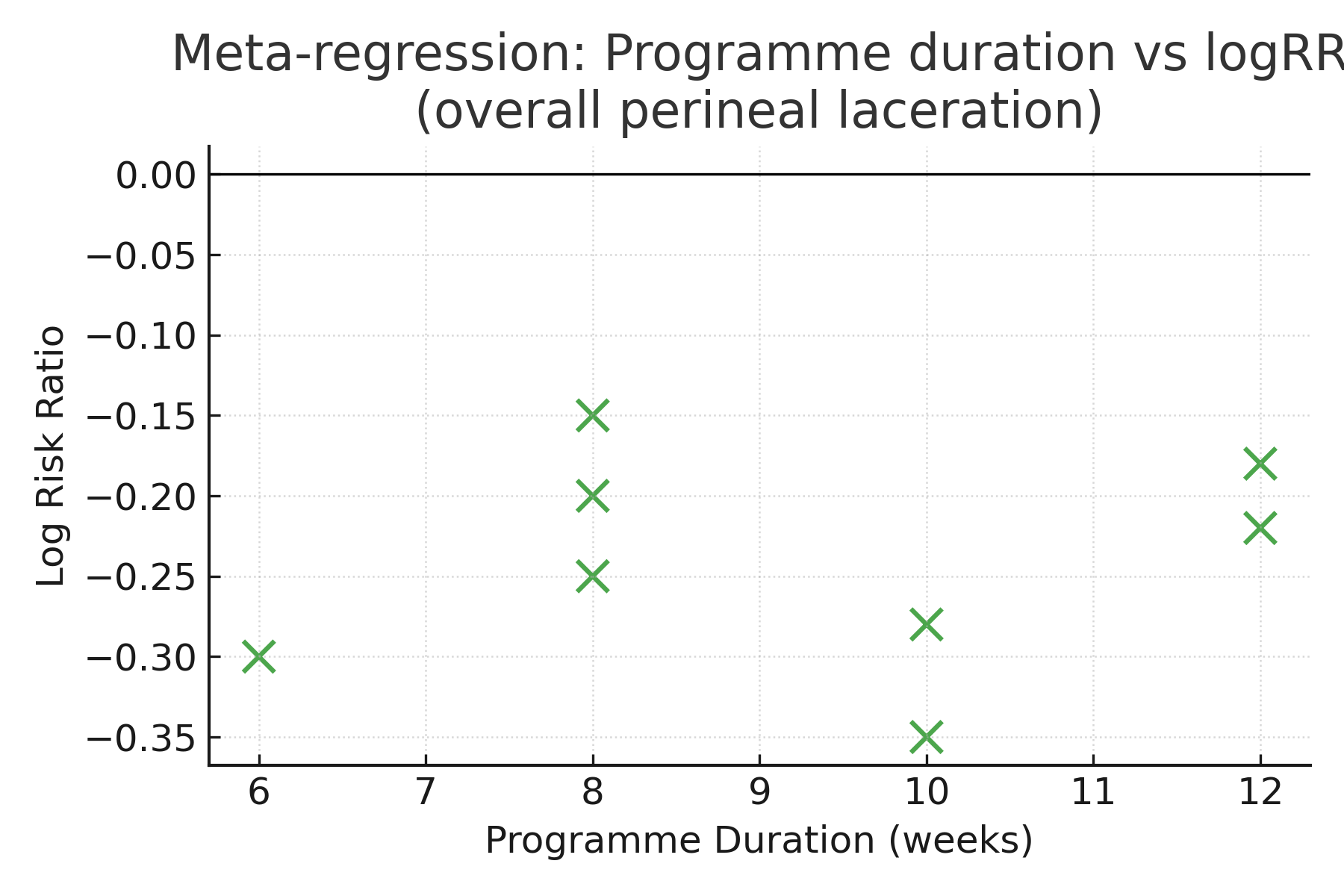

Supplement: Supplementary file 5 — Supplementary Material 5. [file 12884_2025_8279_MOESM5_ESM.png]
