# Supplementary material for: Effects of prenatal yoga on birth outcomes in nulliparous women: a systematic review and meta-analysis of randomized controlled trials
Source: BMC Pregnancy Childbirth. 2025 Dec 10;25:1302. doi: 10.1186/s12884-025-08279-4 (PMC12699887; doi:10.1186/s12884-025-08279-4)
Supplement: Supplementary file 6 — Supplementary Material 6. [file 12884_2025_8279_MOESM6_ESM.docx]

## Table S1. PICO Eligibility Criteria.

| PICO element | Inclusion criteria | Exclusion criteria |
| --- | --- | --- |
| Population | Primiparous pregnant women ≥ 18 y with singleton, term or late‑preterm gestation receiving routine antenatal care | Multiparas; multiple pregnancy; major obstetric complications (e.g., pre‑existing diabetes, hypertension, placenta previa) |
| Intervention | Structured prenatal yoga programme (≥ 4 supervised sessions, any style) initiated during pregnancy | Non‑yoga exercise only; postpartum yoga; programmes where yoga effect cannot be isolated |
| Comparator | Usual antenatal care or non‑exercise control | Active exercise comparators (e.g., Pilates, aerobic classes) |
| Outcomes | Mode of delivery (cesarean, vaginal, instrumental); perineal trauma; pre‑term birth (< 37 weeks); total labour duration | Outcomes unrelated to birth or obstetric parameters |
| Study design | Randomised controlled trial | Observational, quasi‑experimental or uncontrolled studies |
